# Developmental and Anatomical Patterns Of IL-2 Gene Expression *in Vivo* in The Murine Thymus

**DOI:** 10.1155/1993/86096

**Published:** 1993

**Authors:** Julia A. Yang-Snyder, Ellen V. Rothenberg

**Affiliations:** Division of Biology 156-29, California Institute of Technology Pasadena California 91125 USA

**Keywords:** Interleukin-2 (IL-2), interleukin-2 receptor (IL-2R), thymus, *in situ* hybridization, immunohistochemistry, severe combined immunodeficiency (*scid*), T-cell receptor (TcR)

## Abstract

Interleukin-2 (IL-2) is a potent growth factor that mature T lymphocytes synthesize and use as a proliferation signal. Much controversy has arisen concerning whether it is used to drive the extensive proliferation of immature pre-T cells in the thymus. Immature thymocytes acquire the competence to express IL-2 at an early stage, but it has remained uncertain whether they are activated to exercise this competence *in vivo*. Therefore, we have used *in situ* hybridization and immunohistochemistry on serial sections obtained from fetal and adult thymuses of normal C57BL/6 mice and of mice bearing the scid defect to determine where, when, and whether IL-2 is expressed *in vivo*. Our results show a striking spatial and temporal pattern of IL-2 expression in the normal fetal thymus. We detected a burst of IL-2 mRNA accumulation at day 14.5 of gestation, which rapidly decreased by day 15. At day 15, we observed maximal IL-2 protein production that subsequently decreased by day 16 of gestation. Both *in situ* hybridization and immunohistochemical staining revealed an unexpectedly strict localization of IL-2 expressing cells to patches around the periphery of the fetal thymus, creating a previously unrecognized compartment of high IL-2 protein content. IL-2 production in the day-15 fetal thymus appeared to be unaffected by the scid mutation, indicating that this response is likely to be T-cell receptor (TcR)-independent. Several features distinguish the IL-2 induction pattern in the adult thymus from that in the fetal thymus. In the normal adult thymus, IL-2-expressing cells are extremely rare (found at a frequency of 10^-7^), but they are reproducibly detectable as isolated cells in the outer cortex and subcapsular region of the thymus. Unlike the fetal thymic IL-2 producers, the IL-2 producers in the adult thymus are completely eliminated in mice homozygous for *the scid* mutation. This suggests that the IL-2-expressing cells in the normal adult thymus are of a more mature phenotype than the immature, TcR-negative cells that accumulate in the scid adult thymus. Thus, our work demonstrates that two developmentally distinct types of cell interactions induce IL-2 expression *in vivo*: one, a broadly localized interaction in day 14‑15 fetal thymus that is unaffected by *the scid* mutation; the other, a rare event that occurs asynchronously from late fetal through adult life, but which is completely eliminated by the scid defect. These results imply that significant differences exist between the physiological processing of thymocytes in the fetal and postnatal thymic microenvironments.


*Developmental Immunology*, 1993, Vol. 3, pp. 85–102
Reprints available directly from the publisher
Photocopying permitted by license only

© 1993 Harwood Academic Publishers GmbH
Printed in the United States of America

### Developmental and Anatomical Patterns of IL-2 Gene Expression *In Vivo* in the Murine Thymus

JULIA A. YANG-SNYDER and ELLEN V. ROTHENBERG*

*Division of Biology, 156-29, California Institute of Technology, Pasadena, California 91125*

Interleukin-2 (IL-2) is a potent growth factor that mature T lymphocytes synthesize and use as a proliferation signal. Much controversy has arisen concerning whether it is used to drive the extensive proliferation of immature pre-T cells in the thymus. Immature thymocytes acquire the competence to express IL-2 at an early stage, but it has remained uncertain whether they are activated to exercise this competence *in vivo*. Therefore, we have used *in situ* hybridization and immunohistochemistry on serial sections obtained from fetal and adult thymuses of normal C57BL/6 mice and of mice bearing the *scid* defect to determine where, when, and whether IL-2 is expressed *in vivo*. Our results show a striking spatial and temporal pattern of IL-2 expression in the normal fetal thymus. We detected a burst of IL-2 mRNA accumulation at day 14.5 of gestation, which rapidly decreased by day 15. At day 15, we observed maximal IL-2 protein production that subsequently decreased by day 16 of gestation. Both *in situ* hybridization and immunohistochemical staining revealed an unexpectedly strict localization of IL-2 expressing cells to patches around the periphery of the fetal thymus, creating a previously unrecognized compartment of high IL-2 protein content. IL-2 production in the day-15 fetal thymus appeared to be unaffected by the *scid* mutation, indicating that this response is likely to be T-cell receptor (TcR)-independent. Several features distinguish the IL-2 induction pattern in the adult thymus from that in the fetal thymus. In the normal adult thymus, IL-2-expressing cells are extremely rare (found at a frequency of $10^{-7}$), but they are reproducibly detectable as isolated cells in the outer cortex and subcapsular region of the thymus. Unlike the fetal thymic IL-2 producers, the IL-2 producers in the adult thymus are completely eliminated in mice homozygous for the *scid* mutation. This suggests that the IL-2-expressing cells in the normal adult thymus are of a more mature phenotype than the immature, TcR-negative cells that accumulate in the *scid* adult thymus. Thus, our work demonstrates that two developmentally distinct types of cell interactions induce IL-2 expression *in vivo:* one, a broadly localized interaction in day 14–15 fetal thymus that is unaffected by the *scid* mutation; the other, a rare event that occurs asynchronously from late fetal through adult life, but which is completely eliminated by the *scid* defect. These results imply that significant differences exist between the physiological processing of thymocytes in the fetal and postnatal thymic microenvironments.

KEYWORDS: Interleukin-2 (IL-2), interleukin-2 receptor (IL-2R), thymus, *in situ* hybridization, immunohistochemistry, severe combined immunodeficiency *(scid)*, T-cell receptor (TcR).

### INTRODUCTION

T cells, unlike other cell types of the hematopoietic lineage, differentiate in the thymus, where differentiation is accompanied by extensive proliferation, apparently triggered by mitotic signals from the thymic stroma. Thymus-specific lymphostromal interactions are critical for driving T-cell development, yet both the nature of the signals and the molecular basis of their impact remain unknown. Among the

*Corresponding author.

properties acquired and modulated as thymocytes develop is the competence to express "response" genes that drive proliferation in mature T cells, such as the gene encoding the potent growth factor interleukin-2 (IL-2) (McGuire and Rothenberg, 1987; Howe and MacDonald, 1988; Rothenberg et al., 1988; Tentori et al., 1988b; Fischer et al., 1991; Chen and Rothenberg, 1993). Although the inducibility of these genes at certain stages of differentiation is easily demonstrable under artificial conditions *in vitro,* it is not known whether intrathymic signals provide inductive stimuli for these genes *in vivo.* Thus, it is unclear to what extent growth factors such as IL-2 are normally available to contribute to the hormonal microenvironment of the thymus. Similarly, it has remained uncertain whether the characteristic signaling pathways that activate IL-2 expression *in vitro* are utilized in normal thymocyte development *in vivo.*

Recent gene-disruption experiments have formally proven that IL-2 is not required for the generation of normal cell populations in the thymus (Schorle et al., 1991). However, severe perturbations can be induced in thymocyte development, *in vivo* or in organ culture, by anti-IL-2R antibody treatment (Jenkinson et al., 1987; Tentori et al., 1988a; Zuñiga-Pflücker and Kruisbeek, 1990; Zuñiga-Pflücker et al., 1990), by the addition of excess IL-2 (Skinner et al., 1987; Plum and de Smedt, 1988; Waanders and Boyd, 1990), or by the introduction of a transgenic IL-2R$\alpha$ chain with inappropriate species specificity (Kroemer et al., 1991). Thus, the possibility remains that IL-2 production *in vivo* plays some developmental role. Furthermore, studies from our own laboratory and from others have shown that the intrinsic competence to express the IL-2 gene is shared by both mature and immature thymocytes (Howe and MacDonald, 1988; Rothenberg et al., 1990; Fischer et al., 1991). However, it is unknown whether the thymic microenvironment can actually provide signals to induce IL-2 expression in either or both populations of thymocytes. Therefore, IL-2 production (1) could potentially be induced at any of several stages *in vivo* and (2) might cause autocrine or paracrine effects, either early or late in T-cell development. These issues make it instructive to determine the conditions under which developing thymocytes actually exercise their competence to express IL-2 *in vivo.*

Several laboratories have analyzed cytokine expression during fetal thymocyte development *in vivo* as an approach to determine which factors might be present in the thymus during T-cell development. Von Boehmer and coworkers initially reported that IL-2 is secreted as fetal thymocytes mature in organ culture (Kisielow et al., 1985). Subsequent studies have shown that murine embryonic thymocytes harvested at different times during gestation express a variety of cytokine mRNAs, using either *in situ* hybridization to cytocentrifuged preparations of freshly isolated cells (Carding et al., 1989, 1990; Zuñiga-Pflücker et al., 1990) or reverse transcription/polymerase chain reaction (rtPCR) analysis of total RNA samples obtained from fetal thymuses (Montgomery and Dallman, 1991). Most of these studies have focused exclusively on dissociated cell suspensions, making localization and identification of the IL-2-expressing cells within the thymus impossible. On a more general level, these studies have failed to distinguish between the developmental events involved in the fetal ontogeny of the thymus itself and those involved in the maturation of T-cell precursors processed therein. In adult animals, thymocyte development generates a different spectrum of T-cell subtypes than that generated during thymocyte development in the fetus (reviewed in Rothenberg, 1992). This could be attributable either to differences in the thymic environment or to the difference in hematopoietic origins between the precursors that seed the fetal and the postnatal thymus (Ikuta et al., 1990). Thus, it is not clear whether IL-2 production is a universal feature of T-cell development or a peculiarity of the cell types present in the fetal thymus.

In order to examine the spatial and temporal pattern of IL-2 gene expression as induced naturally during T-cell ontogeny *in vivo,* we have used a combination of *in situ* hybridization and immunohistochemical staining of mouse thymus sections isolated at different stages of development. Our findings show different and distinctive patterns of IL-2 gene expression in fetal versus adult thymuses. Additionally, IL-2 gene expression appears to be normal in the fetal thymus but perturbed in the adult thymus of animals that cannot generate mature T cells as a result of the *s*evere *c*ombined *i*mmuno*d*efficiency (*scid*) mutation.

### RESULTS

### Developmental Pattern of IL-2 Gene Expression in the Fetal Thymus as Determined by *In Situ* Hybridization

Figure 1 shows the patterns of IL-2-specific hybridization in the murine fetal thymus at different stages of gestation as defined using 590 nt antisense or sense riboprobes. At day 14, a few highly positive cells were found, mainly in the outer region of the thymus (Fig. 1A). At day 14.5, the outer region of the thymus contained a dramatically increased number of highly positive cells, clustered in distinctive, large patches of hybridization one cell deep that "outlined" the periphery of the tissue (Fig. 1C). At this stage of gestation, no corticomedullary boundary was observed in the thymus; cells appeared equally distributed throughout the organ (Figs. 1C and 1D). Although the majority of sections of day 14.5 fetal thymus did not include any regions of strong hybridization, every thymus examined contained sections exhibiting this striking localization of highly positive cells. By day 15, the percentage of IL-2 mRNA positive cells decreased, and only an occasional highly positive cell was found (6–8 per half lobe equivalent; Fig. 1E). Isolated positive cells were also observed later, at day 18 of gestation, and distinct cortical and medullary regions were identifiable by this time by DAPI staining (data not shown). The remaining IL-2 mRNA positive cells in the fetal thymus at later stages of gestation were still restricted to the cortex and not to the medulla. Thus, highly positive IL-2 mRNA-expressing cells were present at day 14 of gestation in the fetal thymus and some highly positive cells persisted through day 18 of gestation. However, a dramatic peak of highly localized IL-2 gene expression was observed at day 14.5, which decreased abruptly by day 15.

### Pattern of IL-2 Expression in the Fetal Thymus at Different Stages of Development by Immunohistochemistry

To confirm the pattern of IL-2 expression as determined from the *in situ* hybridization data, fetal thymuses isolated at various stages of gestation were also stained with an anti-IL-2 mAb (Fig. 2). At day 14–14.5, weak immunoreactivity was observed (Fig. 2A; data not shown). At day 15, strong staining appeared in the outer region of the tissue (Fig. 2C). This staining pattern was not seen when normal rat IgG was substituted for the anti-IL-2 mAb (Fig. 2D); the IL-2 specificity of the staining reagent was further confirmed by control experiments with excess rmuIL-2 (see below). At day 16, immunoreactivity was still present around the periphery of the thymus, albeit at a reduced level (Fig. 2E), and at day 18, only isolated immunoreactive cells were found in the cortical region. A background of endogenous peroxidase-positive cells became detectable by day 18 (data not shown), but these cells could be distinguished from IL-2 immunoreactive cells by prior blocking and peroxidase quenching (for additional controls, see below). Nonetheless, based on these characteristics, the pattern of IL-2 protein expression, detected by immunohistochemistry, recapitulated the pattern of IL-2 mRNA accumulation as determined by *in situ* hybridization. Additionally, maximum IL-2 protein expression was observed 12 hr following maximum IL-2 mRNA accumulation.

### V$\gamma$3 and CD16 Expression versus IL-2 Expression in Day 15 Fetal Thymus as Determined by Immunohistochemistry

IL-2 has been implicated in the growth of V$\gamma$3$^+$ T cells and natural killer (NK) cells from the fetal thymus *in vitro* (Skinner et al., 1987; LeClerq et al., 1990). Therefore, we were interested in determining whether intrathymic IL-2 was associated with the generation or expansion of particular subsets of T cells or NK cells in the fetal thymus *in vivo.* By day 15 of gestation, almost all of the rare thymocytes expressing surface TcR specifically utilize the V$\gamma$3 segment in TcR gene rearrangements (Havran and Allison, 1988). Likewise, the majority of cells in the fetal thymus at day 15 of gestation express CD16 (FcRII/III; Rodewald et al., 1992), a marker that persists preferentially on pre-NK and NK cells. We used a V$\gamma$3-specific (536) or CD16-specific (2.4G2) mAb along with the IL-2-specific mAb for immunohistochemical staining of adjacent serial sections of day 15 fetal thymus to determine the relative location of V$\gamma$3$^+$ and CD16$^+$ cells with respect to IL-2-producing cells (Fig. 3). At this stage of gestation, the majority of the cells in the fetal thymus were cytoplasmic CD3$\varepsilon^+$ (data not

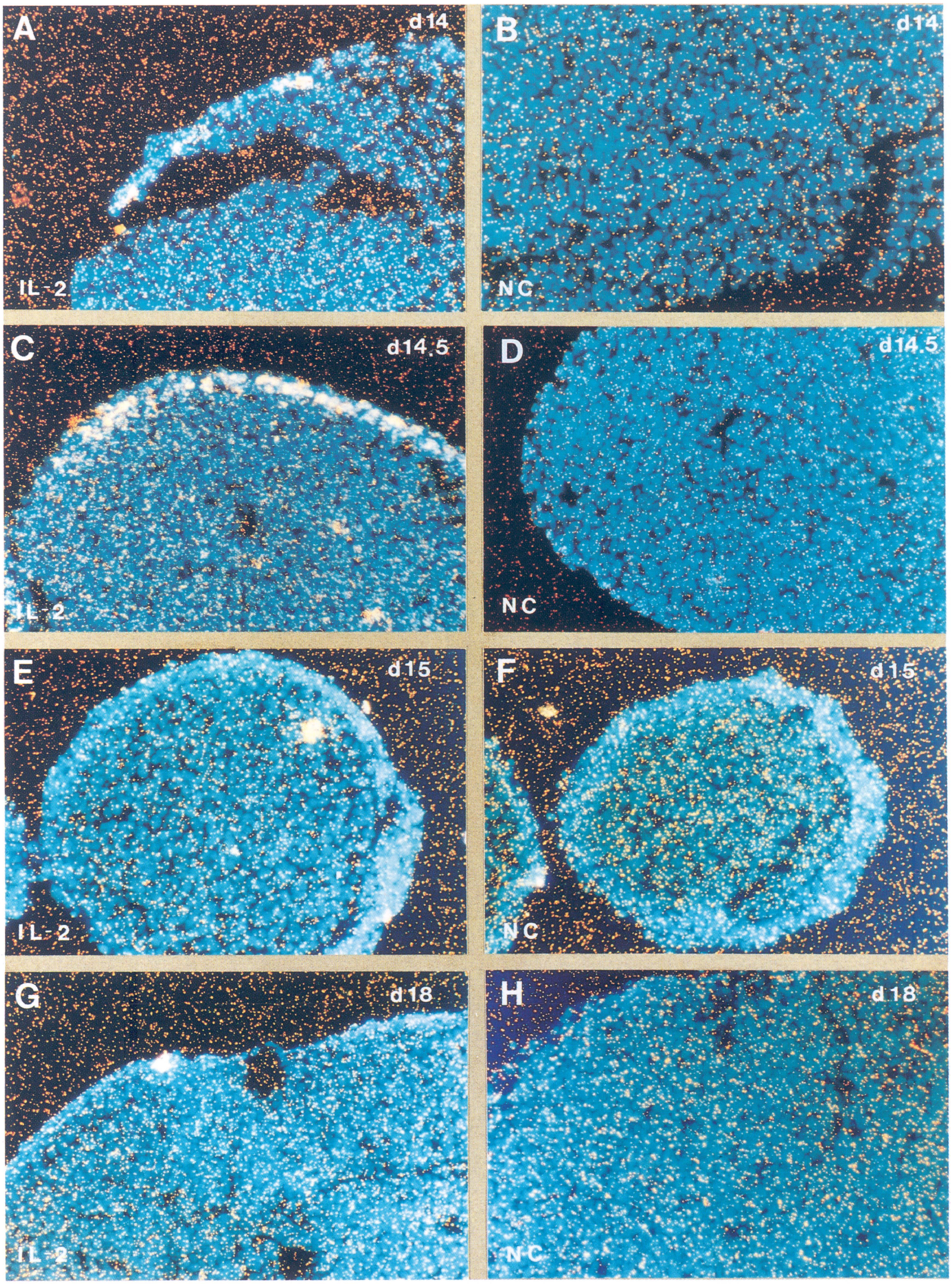

A
d14
IL-2
B
d14
NC
C
d14.5
IL-2
D
d14.5
NC
E
d15
IL-2
F
d15
NC
G
d18
IL-2
H
d18
NC

FIGURE 1.

shown). $CD16^+$ cells were detected in a heterogeneous distribution throughout the fetal thymus. $V\gamma3^+$ cells were also detectable in a similar distribution (Fig. 3), but were substantially less abundant than $CD16^+$ cells. Neither cell type was concentrated in the peripheral region of the lobe. Thus, these results gave no clear evidence for colocalization of IL-2-producing cells with either $V\gamma3$ or CD16 expression (see Discussion).

### IL-2 Expression in the *Scid* Day 15 Fetal Thymus

Mice homozygous for the *scid* mutation fail to rearrange their TcR genes normally, causing a developmental arrest that blocks the production of all classes of $TcR^+$ thymocytes (Bosma and Carroll, 1991). In order to determine whether the *scid* defect affected IL-2 expression in the fetal thymus, we used immunohistochemistry to detect IL-2-producing cells in the *scid* day 15 fetal thymus (Figs. 2I to 2L). *Scid* day 15 fetal thymuses appeared normal in terms of the intensity and localization of IL-2 immunoreactivity (Fig. 2I). This staining was also shown to be IL-2-specific. When an excess of exogenous recombinant IL-2 was added, IL-2 immunoreactivity was abolished (Figs. 2K and 2L); as a control, addition of recombinant IL-4 had little effect (data not shown). Likewise, the gross architecture and size of the *scid* day 15 fetal thymus appeared to be normal in terms of cell density and number (data not shown). *In situ* hybridization of *scid* day 15 fetal thymus sections revealed that a similar number of highly labeled IL-2 mRNA positive cells were present as compared to the normal counterpart (data not shown). Hence, at this stage of gestation, TcR rearrangement and expression are not prerequisites for IL-2 expression *in vivo.*

Analysis of IL-2R$\alpha$ expression confirmed the lack of gross perturbation of thymocyte development in *scid* mice at this age. When sections were stained with a rat anti-mouse IL-2R$\alpha$ mAb, normal and *scid* day 15 fetal thymuses were similar with regard to the number and distribution of IL-2R$\alpha^+$ cells in the thymus (Figs. 4A and 4B). By contrast, differences were readily apparent when comparing the IL-2R$\alpha$ staining pattern of the normal versus *scid* adult thymus. The overwhelming majority of the cells in all regions of the *scid* adult thymus are IL-2R$\alpha^+$, as compared with the irregular clusters of IL-2R$\alpha^+$ cells in the cortex of the normal tissue (Figs. 4C and 4D). This aberrant IL-2R$\alpha$ staining pattern of the *scid* adult thymus convincingly showed the developmental arrest-blocking progression beyond the IL-2R$\alpha^+$ immature stage. These staining results indicated that the *scid* mutation did not severely affect the control of IL-2 or IL-2R$\alpha$ expression in the fetal thymus, in spite of its severe developmental effects observed in the adult thymus.

### *In Situ* Hybridization of Normal Adult Thymus Sections

In previous analyses of cytocentrifuged preparations of adult thymus cell suspensions, we had failed to detect any cells expressing IL-2 mRNA (McGuire and Rothenberg, 1987; Rothenberg et al., 1990). Ribonuclease protection assays failed to detect any IL-2 transcripts in samples of up to 100 $\mu$g of adult thymus total RNA, roughly equivalent to the amount of RNA isolated from half a thymic lobe (400 $\mu$g total RNA/thymus; data not shown). However, the results of four independent experiments confirmed the presence of extremely rare, highly labeled IL-2 mRNA-positive cells in the thymus of 4-week-old mice. Approximately 160 sections hybridized alternately with each strand of the 430-nt probe were analyzed per experiment (approximately 320 sections examined overall, corresponding to a thymus lobe; i.e., a half lobe equivalent for each probe). Highly labeled cells were found only in sections that had been hybridized with the antisense riboprobe. These IL-2-positive cells were reproducibly found in the outer cortex and subcapsular region of the thymus (Fig. 5A); the cortical versus medullary regions of the thymus were distinguished by higher or lower cell density within the section, as shown by DAPI staining (Figs. 4A and 4B). Surprisingly, of all the IL-2-

FIGURE 1. (See Colour Plate VI at the back of this publication). *In situ* hybridization to sections of normal thymuses from days 14 to 18 of gestation for IL-2 expression, using either a $^{35}S$-labeled, 590-nt IL-2 antisense RNA probe (IL-2; Figs. 1A, 1C, 1E, 1G) or the complementary sense strand (NC; Figs. 1B, 1D, 1F, 1H). Samples were counterstained with DAPI and observed under dark field microscopy. 14-day exposure; magnification ×200.

TABLE 1
Titration of 430-nt 3'-IL-2 Probe by Ribonuclease A, T1 Protection Using Total RNA Isolated from Induced EL4 Cells

| % induced EL4[a] | % IL-2 mRNA$^+$ by *in situ*[b] | No. copies IL-2 protected[c] | No. copies IL-2 per avg. cell[d] | No. copies IL-2 per induced cell[e] | No. copies IL-2 per mRNA$^+$ cell[f] |
|---|---|---|---|---|---|
| 1 | 0.64 | $1.5 \times 10^6$ | 1.5 | 150 | 234 |
| 2 | 1.4 | $2.9 \times 10^6$ | 2.9 | 145 | 207 |
| 5 | 3.4 | $7.1 \times 10^6$ | 7.1 | 140 | 206 |
| 10 | 8.1 | $1.8 \times 10^7$ | 18 | 180 | 222 |
| 20 | 13.9 | $3.3 \times 10^7$ | 33 | 165 | 240 |
| 50 | 35.0 | $7.9 \times 10^7$ | 79 | 160 | 229 |
| 100 | 74.0 | $1.7 \times 10^8$ | 170 | 170 | 230 |
| The average number of copies IL-2 per IL-2 mRNA$^+$ cell=224±18 | | | | | |

[a]EL4 cells induced for 5 hr with calcium ionophore and phorbol ester were mixed with uninduced EL4 cells at the percentages indicated (a total of $5 \times 10^6$ viable cell equivalents per sample).

Total RNA was extracted according to the method described by Chomczynski and Sacchi (1987).

One-fifth of each sample (or RNA from $1 \times 10^6$ cell equivalents) was used per protection. No protection was observed above background using total RNA isolated from uninduced EL4 cells.

[b]Identical samples of cells were processed as described in Materials and Methods and analyzed by *in situ* hybridization. Signals from at least 5000 cells were analyzed per sample.

[c]$$\text{No. copies protected} = \text{conversion factor} \times \frac{\text{phosphor imager value for protected product}}{\text{cpm undigested probe used per protection}} \times \frac{\text{ng probe used per protection}}{\text{molec. wt. protected product}} \times 6 \times 10^{23} \text{ molecules per mole}$$

where the conversion factor = $\frac{\text{(liquid scintillation cpm}}{\text{phosphor imager value)}}$, determined for known amounts of undigested probe.

[d]$$\frac{\text{No. copies IL-2 protected}}{1 \times 10^6}$$

[e]$$\frac{\text{No. copies IL-2 per avg. cell}}{\text{\% ind. EL4} \times 10^{-2}}$$

[f]$$\text{No. copies IL-2 per ind. cell} \times \frac{\text{\% induced EL4}}{\text{\%IL-2 mRNA}^+ \text{ by } in\ situ}$$

least 100-fold for immature thymocytes at all stages leading up to the $CD3^-CD4^-CD8^-$ (TN) IL-$2R\alpha^+$ stage of development (see Fig. 4) (Bosma and Carroll, 1991). If the IL-2 mRNA-positive/immunoreactive cells in the thymus of normal animals were of an immature phenotype, they should be present in greater numbers in the *scid* adult thymus than in the normal counterpart. On the other hand, if these cells had matured past the stage of TcR gene rearrangement, they should be depleted in the *scid* adult thymus. *In situ* hybridization revealed that IL-2 mRNA-positive cells were conspicuously absent from the *scid* adult thymus; both antisense and sense probes gave identical results in all sections analyzed (Figs. 5D and 5E). The absence of hybridization in the *scid* adult thymus samples was not due to artifactual degradation of probes hybridized to the *scid* thymus sections, because alternate sections hybridized with IL-2R$\alpha$ antisense probes yielded strong signals (Fig. 5F), consistent with the results obtained from direct immunofluorescent staining (Fig. 4D).

For direct quantitative comparison, hybridization of *scid* thymus sections were carried out in parallel with hybridization of normal thymus sections (Fig. 6). On average, fewer sections per *scid* thymus were analyzed than per normal thymus because *scid* adult thymuses were substantially smaller than normal adult thymuses (*scid* thymuses, which lack all TcR$^+$ cell types, contain approximately 1/100 the number of viable cells found in normal thymuses; data not shown). Due to the reduced cell numbers in the *scid* adult thymus, the frequency of IL-2-expressing cells could not be proven to be lower in the *scid* adult thymus than in the normal adult thymus on a per cell basis. Nonetheless, the depletion of IL-2 mRNA-positive cells per lobe in the *scid* thymus was highly significant (Fig. 6).

The absence of cells producing IL-2 protein in the *scid* adult thymus was difficult to confirm by immunohistochemistry due to a high background of endogenous, peroxidase-positive cells that were "reactive" in the absence of specific antibody, even after extensive $H_2O_2$/peroxidase quenching (data not shown). Nevertheless, based on the unambiguous *in situ* hybridization data, it

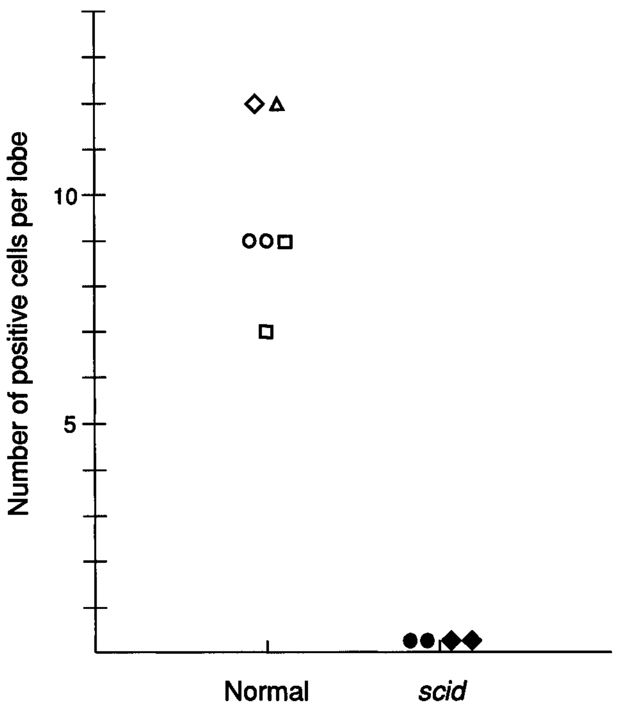

Average Number of Sections Hybridized with Either Strand: Normal=162
*scid* = 80
Average Number of Sections Analyzed per Experiment: Normal=324
*scid* =160

FIGURE 6. Compiled data of *in situ* hybridization for IL-2 expression in adult thymus from normal and *scid* mice. Adult thymus sections from normal and *scid* animals (open and shaded points, respectively) were analyzed by *in situ* hybridization with the 430-nt antisense or sense IL-2 RNA probe. Approximately half a thymic lobe was hybridized with each probe. The results using the antisense probe were compiled from four independent experiments. No signals were detected with the sense probe (data not shown). Normal and *scid* sections hybridized and analyzed in parallel are denoted with identical shapes (circles, diamonds). Positive cells were considered as having greater than 10 silver grains. The average, total numbers of sections analyzed per normal and *scid* thymus were 320 and 80, respectively, corresponding to complete thymic lobes. Slides were exposed for 21 days.

appeared that IL-2 mRNA-positive cells in the normal adult thymus were very likely to be of a more mature phenotype than the developmentally arrested thymocytes found in the *scid* adult thymus.

### DISCUSSION

Our results show that IL-2 expression is induced in thymocytes by intrathymic signals *in vivo*. This expression is subject to complex developmental regulation that constrains the possible roles of IL-2 as a mediator of signals. On the other hand, the induction of IL-2 itself serves as a probe for specific cell–cell interactions, which reveal surprising differences between the fetal and adult thymus.

### Restricted Activation of IL-2 Expression in the Fetal Thymus

Our results show that the day 15–16 fetal thymus includes distinct, substantial zones of low and high regional IL-2 protein concentration, in good agreement with the results of Waanders (1990). Twelve hours prior to maximal protein production, a burst of IL-2 message accumulation is observed in cells restricted to the identical area where IL-2 protein will be found, namely, the periphery of the fetal thymus. The combined *in situ* hybridization and immunohistochemical staining data reported here for day 14.5–15 fetal thymus show that only a minority of the cells in the fetal thymus were expressing IL-2 message or protein at the given time points. This interpretation is not in complete agreement with that of Zuñiga-Pflücker et al. (1990), who reported that 50–60% of fetal thymocytes expressed IL-2 mRNA at day 15 gestation, based on *in situ* hybridization to cytocentrifuged samples. The disparity in percentage of IL-2 mRNA-positive cells could be due to any of several factors. Because the kinetics of IL-2 expression are clearly transient, our time points might have missed the true peak. Alternatively, the timing of the actual impregnation of the mice could be affected by the setting of the 12-hr light–dark cycle in the respective mouse colonies, leading to apparent shifts in kinetics. Also, in principle, there could be a lower threshold of detection for *in situ* hybridization to cytocentrifuged cell suspensions than to tissue sections. Finally, as Zuñiga-Pflücker et al. (1990) did not subdivide categories of IL-2 mRNA-positive cells based on silver grain count, they might have included cells that we would not consider to be unequivocally IL-2-positive. However, our immunohistochemical staining data support our *in situ* hybridization results, indicating that only a highly localized minority population express IL-2 protein that is detectable at days 15–16 of gestation. Thus, according to these staining results, if 50–60% of cells do express an IL-2 message at the time of our terminology) grown in moderate amounts of IL-2 (Rodewald et al., 1992). Furthermore, transgenic mice constitutively expressing IL-2 and IL-2R$\alpha$ chain show great preferential expansion of NK cells (Ishida et al., 1989). Based on these data, the generation of either of these cell types in the fetal thymus *in vivo* could be positively regulated by the local concentration of IL-2. However, when we examined V$\gamma$3, CD16, and IL-2 expression in the day 15 fetal thymus by immunohistochemical staining, we were unable to show a relationship between the localization of V$\gamma 3^+$ or CD16$^+$ cells and IL-2 producers (Fig. 4). Although we cannot formally disprove the possibility that V$\gamma 3^-$ or CD16$^-$ precursors of either cell type respond to IL-2, these results make it unlikely that IL-2 is limiting for the generation of V$\gamma 3^+$ T cells or CD16$^+$ cells (presumptive T/NK precursors) in the fetal thymus *in vivo*. The targets for the burst of fetal IL-2 production therefore remain undefined.

In contrast to the results obtained from day 14.5–15 fetal thymuses, IL-2-expressing cells are extremely rare in the adult thymus with a frequency on the order of $10^{-7}$, as determined by both *in situ* hybridization and immunohistochemical staining. Any cells responding to IL-2 in the adult thymus *in vivo* would most likely need to be in close proximity to these IL-2 producers, based on the minimal diffusion of IL-2 protein observed in the cortex. Unlike IL-2 production in the fetal thymus, no continuous zones of high IL-2 concentration are found in the adult organ. Furthermore, IL-2 may be available in these local sites for only a limited time. Our data suggest that the steady-state frequency of IL-2-expressing cells reflects the asynchronous activation of different rare cells. In the adult thymus, cells producing IL-2 protein were detected at an approximately threefold higher frequency than cells expressing IL-2 mRNA at any one time. The IL-2 mRNA is known to be labile (Shaw et al., 1988; Lindsten et al., 1989), and our results with the fetal thymus indicate that IL-2 protein can persist in the thymus at least 1 day longer than IL-2 mRNA. Thus, we interpret the excess of IL-2 immunoreactive cells over IL-2 mRNA-positive cells as evidence that IL-2 gene induction in the adult thymus is also transient but asynchronous; that is, that most cells synthesizing IL-2 protein have already shut off synthesis of the short-lived IL-2 mRNA. When the resulting limited availability of IL-2 is compared with the 10–20% of thymocytes that are actively in cycle (Penit, 1986; Boyer et al., 1989), it is clear that thymocyte-derived IL-2 is grossly inadequate to drive the extensive proliferation in this organ.

Our results support the finding that transgenic mice homozygous for an IL-2 gene disruption did not make IL-2 and yet generated all major thymocyte populations in normal numbers (Schorle et al., 1991). However, the extreme rarity of IL-2-rich "niches" in the normal adult thymus raises a caveat about the interpretation of the gene disruption results. If IL-2 were normally required as a growth factor for a particular subset of cells in the thymus, then its targets must represent a very minor fraction of cells in the cortex, the deletion of which might not drastically affect thymocyte population dynamics as a whole. Thus, an effect of IL-2 on some minor or transient branch of the T-cell lineage cannot be ruled out.

### Developmental Significance of IL-2 Induction *In Vivo*: IL-2 as an Indicator of a Rare Activation Event

What may be the most telling aspect of our data is the access they provide into the intrathymic activation events that are detected as the induction of IL-2 gene expression in the murine thymus *in vivo*. These activation events depend both on the delivery of the appropriate signal from the thymic microenvironment and on the developmental status of the responding thymocyte (Howe and MacDonald, 1988; Rothenberg et al., 1990; Fischer et al., 1991). Little is known about the biochemical basis of thymic lymphostromal interactions, and key events in which activation signals are delivered have been more commonly inferred than demonstrated. On the other hand, the IL-2 induction response *in vitro* is relatively well characterized, both in terms of cytoplasmic signaling cascade and the transcription factors that are mobilized in the activated cells. Thus, our results provide strong evidence that this particular cascade of responses is indeed triggered *in vivo* in particular cells in the fetal and postnatal thymus.

As both immature (TcR$^-$) and mature (TcR$^{high}$) thymocytes can in principle make IL-2, the intrathymic IL-2 production observed could represent a response either of an immature cell, presumably to differentiating signals, or of a mature cell (possibly to antigen). We have utilized two complementary kinds of evidence to reach a provisional conclusion. One is the intrathymic location of IL-2-expressing cells: In postnatal mice, the cortex is a zone of differentiation and precursor expansion, and the medulla is a domain where mature thymocytes accumulate and encounter circulating cells and antigen. The other relates to the effect of the *scid* mutation on intrathymic IL-2 expression. By preventing correct TcR gene rearrangement, this mutation blocks all TcR-expression-dependent events in T-cell development while permitting those that precede TcR gene rearrangement. The results of our analysis suggest that both fetal and postnatal cells that make IL-2 in the thymus are responding to developmental signals, rather than environmental antigen, but that distinct, nonequivalent cell types are responding to these signals in the fetal versus postnatal thymus.

To elaborate, the fetal thymocytes that make IL-2 are unaffected by the *scid* mutation, even though the *scid* mutation effectively blocks the generation of fetal $TcR^+$ thymocytes. Therefore, the activation signal that induces IL-2 expression in fetal thymocytes is likely to be delivered to cells at a stage upstream of the *scid* arrest point, that is, prior to TcR expression. This interpretation is supported by the failure of IL-2-expressing cells to colocalize with TcR-expressing cells in the day 15 fetal thymus (i.e., $V\gamma3^+$ cells). By contrast, since the *scid* mutation does block the appearance of all IL-2-expressing cells in postnatal thymus, it is probable that postnatal thymocytes normally receive an IL-2-inducing signal *downstream* of the *scid* arrest point, as discussed further below. Thus, in the context of their own developmental lineage, the postnatal IL-2 expressors are likely to be more advanced than the fetal IL-2 expressors. However, the cortical location of the postnatal IL-2 producers argues against any possibility that they have completed their intrathymic processing. Instead, the signal that induces IL-2 expression is apparently delivered while cells are still in the differentiative domain of the thymus.

Successful induction of IL-2 expression *in vivo* indicates not only that thymocytes have the capacity to express IL-2 and that the thymic stroma has the capacity to deliver triggering signals, but also that the signal is matched to satisfy the activation requirements for the particular classes of thymocytes present. The failure of adult *scid* thymocytes to activate IL-2 expression could be caused by defects in any of these aspects. However, our *in vitro* studies (Chen and Rothenberg, 1993; Rothenberg et al., submitted) clearly show that the *scid* thymus is significantly *enriched* for cells with the competence to express IL-2, provided that a certain set of triggering conditions are used *in vitro.* Therefore, either a lack of inductive signal or a mismatch between the available signals and the requirements of the developmentally arrested cells seems to be implicated. One possible explanation for the failure of cells in the *scid* adult thymus to express IL-2 *in vivo* would be that the *scid* thymic microenvironment (i.e., the nonlymphoid component of the thymus) does not develop properly, due to the lack of feedback interactions from mature thymocytes. Indeed, there is some evidence for architectural abnormality of the *scid* thymus (Shores et al., 1990, 1991; van Ewijk, 1991). However, the main components reported to be reduced or absent in the *scid* thymus are medullary epithelium, and as previously noted, the induction of IL-2 expression that we detect in the normal postnatal thymus is never associated with the medulla. Thus, another explanation for the inability of cells in the *scid* adult thymus to express IL-2 *in vivo* seems more plausible, namely, that the *scid* mutation directly prevents the maturation of thymocytes themselves to the stage where they can respond to those IL-2 inductive signals generated by the postnatal thymic cortex. In this case, responsiveness to IL-2-inducing signals in the adult thymus *in vivo* would be at least indirectly dependent on successful TcR gene rearrangement. Whether the TcR itself is implicated in the triggering event remains to be determined. We are currently in the process of defining the signals that induce IL-2 gene expression in the postnatal thymus *in vivo.*

### Conclusions

To summarize, our work shows that IL-2 is expressed in the fetal and adult thymus of normal mice *in vivo.* Although the pattern of IL-2 gene expression differs in the fetal versus adult thymus, IL-2 expression is highly localized during both times in development. In the fetal thymus, high expression of IL-2 is synchronous and transient. Surprisingly, fetal thymic expression of IL-2 *in vivo* is not disrupted by the *scid* mutation, suggesting that normal TcR expression is not a requisite for IL-2 gene expression in the cell types present in the fetal thymus. On the other hand, IL-2 gene expression appears to be dependent upon TcR gene expression in the adult thymus, because the *scid* mutation abrogates the appearance of IL-2-expressing cells in the adult thymus. Finally, our results indicate that IL-2 is available to cells in highly restricted areas of the thymus during development. Taken together, this work implies that TcR-independent and TcR-dependent interactions are made within the thymus at different stages of development that, in turn, provide the proper inducing signal(s) in resident cells for IL-2 expression *in vivo*. Likewise, differences between the fetal and postnatal thymic microenvironments were clearly evident, both in terms of IL-2 protein content and in terms of the subsets of thymocytes induced to express IL-2 therein. The nature of the responsive and responding cells, and the criteria by which they are distinguished from others in the same stage of differentiation are intriguing issues for further investigation.

### MATERIALS AND METHODS

### Mice

C57BL/6 Tla$^+$ and C.B-17-*scid* animals were bred and maintained in our own facility. Thymuses from embryos at various stages of gestation were obtained from females using timed matings. The appearance of a vaginal plug was designated as day 0 and on this day the females were separated from the males. Young "adult" animals were used at 4 weeks of age. All *scid* animals were maintained in an Isotec flexible film isolater (Indianapolis, Indiana) without antibiotic treatment, except those used for timed matings that were kept outside the isolator.

### Removal and Sectioning of Tissues

Fetal thymuses were removed from embryos every 12 hr on and following day 14 of gestation (pregnant females were sacrificed at 10:00 A.M. and 10 P.M.) and were immediately embedded in Tissue-Tek O.C.T. compound (Miles Inc., Kankakee, Illinois). Thymuses removed from adult animals were frozen in n-pentane/dry ice and stored at –70°C and embedded in O.C.T. prior to sectioning. For *in situ* hybridization, 6–8-μm serial cryosections were collected onto poly-L-lysine-coated glass slides, dried briefly, fixed in freshly made 4% paraformaldehyde in PBS for 1–4 min, rinsed in PBS and stored in 70% ethanol at 4°C. Alternatively, rinsed sections were stored with desiccant at –70°C and transferred to ethanol at 4°C, 3–7 days prior to hybridization. For sensitivity controls/hybridization standards, the murine thymoma EL4 was induced to express IL-2 by culturing the cells in the presence of the calcium ionophore A23187 (70 nM) and phorbol myristate acetate (PMA; 17 nM, 10 ng/ml) for 5 hr in RPMI 1640 (Irvine Scientific, Santa Ana, California) based complete medium supplemented with 5% fetal bovine serum (v/v) as described elsewhere (Chen and Rothenberg, 1993). Following induction, cells were harvested, mixed with uninduced cells at a known ratio of induced-to-uninduced cells, embedded in O.C.T., sectioned, fixed, and stored accordingly. For immunofluroescence and immunohistology, 6–8-μm cryosections were collected onto 3-aminopropyltriethoxysilane (Fluka, Ronkonkoma, New York) coated slides, air dried for approximately 1 hr, fixed in cold acetone for 10 s and stored with desiccant at –20°C. For activated lymph node samples, adult mice (8–12 weeks) were immunized by hind foot pad injection using Freund's complete adjuvant. Popliteal, inguinal, and pericardial lymph nodes were removed 8 days after immunization and tissues were processed as described before.

### Hybridization Probes

All IL-2 probes used for *in situ* hybridization were IL-2 cDNA fragments cloned into the polylinker of the pGEM-1 and -2 plasmids (Promega, Madison, Wisconsin) using standard procedures. To ensure IL-2-specific hybridization, RNA probes did not include the CAG-repeat located in the 5'-region of the IL-2 cDNA. Two IL-2 RNA probes were used; a 590-nucleotide (nt) probe gave a strong signal, and a 430-nt AT-rich probe (contained within the 590-nt probe) gave extremely low backgrounds. For the 590 nt probe, a 750-base pair (bp) PstI-AccI fragment derived from the full-length IL-2 cDNA (DNAX, Palo

Alto, California) was subcloned into pGEM-1 and -2 (pCmIL2-1, -2). For the 430-nt probe, a 430-bp HindIII-AccI fragment derived from the same full-length cDNA clone was also subcloned into pGEM-1 and -2 (pmIL2.3-1, -2; McGuire and Rothenberg, 1987). The pCmIL-2 plasmids were linearized with PvuII and the pmIL2.3 plasmids were linearized with HindIII. The IL-2R$\alpha$ probes were derived from two IL-2R$\alpha$ cDNA fragments (Miller et al., 1985), a 5' 410-bp PstI fragment and an internal 460-bp TaqI-PvuII fragment. Both were subcloned into pGEM-1 and were linearized with HindIII. $^{35}$S-labeled sense or antisense transcripts were synthesized using T7 RNA polymerase as described previously (McGuire and Rothenberg, 1987; McGuire et al., 1988). Transcription products were resuspended in 200 $\mu$l of 100 mM DTT and 0.1% SDS in 10 mM Tris, 1 mM EDTA, pH 7.4, and stored at –70°C prior to use. All steps in the procedure were carried out using solutions made with diethylpyrocarbonate-treated water and RNase-free glass- and plasticware.

### *In Situ* Hybridization

For each *in situ* hybridization experiment, alternate serial sections were hybridized with antisense and sense (negative control, "NC" in figures) riboprobes. Cells were only considered positive where the signal with the antisense probe clearly exceeded the sense-strand background. Hybridization was carried out with modifications to a protocol described previously (Rothenberg et al., 1990). The hybridization buffer described previously was modified by the addition of 4 mM vanadyl ribonucleoside complexes, and hybridization was carried out for 8–12 hr. Following hybridization, cover slips were soaked off in 4×SSC. Unhybridized probe was removed with three washes in 50% formamide, 2×SSC, 10 mM DTT, and 1% 2-ME at 50°C (1 hr each). Slides were briefly rinsed in 2×SSC, transferred to digestion buffer (0.5 M NaCl, 10 mM Tris-HCl, pH 7.4, 1 mM EDTA) containing 40 $\mu$g/ml RNase A and 40 U/ml RNase T1 and digested at 37°C for 30 min, after which slides were transferred to digestion buffer with 20 mM DTT and 1% 2-ME and incubated at 37°C for an additional 30 min. Slides were subsequently washed overnight in 50% formamide, 2×SSC, 10 mM DTT, 1% 2-ME at 45°C and/or in 2×SSC, 1 mM DTT, 1% 2-ME at 45°C for 30 min. In each case, the final wash was in 0.1×SSC, 0.1% 2-ME at room temperature for 20 min. Slides were dehydrated in 30, 50, 70, and 95% ethanol containing 0.3 M ammonium acetate, dried for 1 hr and dipped in NTB-2 emulsion (Eastman Kodak Co., Rochester, New York) diluted at a ratio of 2 to 1 with 0.9 M ammonium acetate. Slides were exposed for 12–21 days, developed at 13°C for 3 min in GBX developer (Eastman Kodak Co.), briefly rinsed in 2% acetic acid, and fixed for 7 min in Ektaflo fixer (Eastman Kodak Co.). Counterstaining was done with 250 $\mu$g/ml 4,6-diamidino-2-phenylindole (DAPI) at room temperature for 1–2 hr or with hematoxylin and eosin for 2 min and cover slips were mounted using Permount (Fisher Scientific, Fair Lawn, New Jersey). Dual dark field and fluorescence microscopy were used for analysis.

### Antibodies

The rat anti-mouse IL-2 mAb, S4B6, was obtained commercially at a concentration of 1 mg/ml (Pharmingen, San Diego). Normal rat IgG was reconstituted at a concentration of 20 mg/ml (Miles Inc., Kankakee, Illinois). Immunoselect phycoerythrin (PE)-conjugated rat anti-mouse CD25 was purchased from Gibco BRL (Gaithersburg, Maryland), and biotinylated hamster anti-mouse CD3$\varepsilon$ (145-2C11) and fluorescein (FITC)-conjugated V$\gamma$3 TcR (536) mAbs were purchased from Pharmingen. Biotinylated rabbit anti-rat IgG or goat anti-hamster IgG (both mouse-absorbed) were obtained from Vector (Burlingame, California) or CALTAG Laboratories (San Francisco), respectively. The hybridoma 2.4G2 (rat anti-mouse CD16; FcRII/III) was grown in Fetal Clone II (Hyclone Sterile Systems, Logan, Utah) and pooled culture supernatants were used neat for staining.

### Immunohistochemistry

The immunohistochemical staining protocol was adapted from procedures described by Farr et al. (1990) and Bogen et al. (1991). Sections were warmed to room temperature, fixed in cold acetone for an additional 2 min, transferred to 1% formaldehyde in PBS for 1 min, and washed in PBS for 5 min. Sections were blocked with 50 $\mu$g/ml avidin in PBS, followed by treatment with

250 μg/ml D-biotin in PBS, each for 20 min. Sections were then washed in PBS/0.025% sodium azide ($NaN_3$) and incubated with 2% normal mouse serum (Cappel/Organon Teknika Co., Durham, North Carolina) and 2% normal rabbit serum in PBS/1% BSA/0.025% $NaN_3$ in a humidified chamber at room temperature for 30 min. After every subsequent incubation, sections were washed with PBS/$NaN_3$. Following blocking steps, sections were incubated with 10 μg/ml primary antibody or normal rat IgG in PBS/BSA/$NaN_3$ at room temperature for 1 hr. Sections were then incubated with 10 μg/ml biotinylated secondary staining reagent (mouse absorbed) in PBS/BSA/$NaN_3$ at room temperature for an additional hour. Subsequently, sections were refixed in 2% paraformaldehyde in PBS for 10 min and quenched with 0.6% $H_2O_2$ in methanol for 15 min. Following extensive washing in PBS, staining was detected using an avidin-biotin-horseradish peroxidase complex (Vectastain Elite ABC; Vector Laboratories) and diaminobenzidine (DAB) as the enzymatic substrate (as described in the Vectastain protocol). Blocking experiments were performed using recombinant murine IL-2 or IL-4 purchased from Genzyme (Boston) and were carried out by preincubating the primary antibody with an excess of recombinant lymphokine (1000 U/ml) at room temperature for at least 1 hr prior to staining. Nickel/cobalt-enhanced staining was achieved using 30 mg $NiCl_2$ and 30 mg $CoCl_2$ per 10 ml DAB: $H_2O_2$ developing solution.

### Direct Immunofluorescence

Staining procedure was modified from a protocol provided by Calbiochem (La Jolla, California). Sections were warmed to room temperature, fixed in cold acetone and briefly in 1% formaldehyde in PBS, and washed in PBS. Sections were then blocked with 2% normal mouse serum in PBS/BSA/$NaN_3$. Following a PBS/$NaN_3$ wash, sections were incubated with 6–10 μg/ml primary antibody in PBS/BSA/$NaN_3$ in a humidified chamber for 1 hr. Sections were washed again with PBS/Na azide. Stained sections were covered with 2–4 drops of Fluorosave Reagent (Calbiochem, La Jolla) and cover slips were mounted and sealed with clear nail polish.

### Ribonuclease Probe Protection

Total RNA from samples containing known percentages of induced and uninduced EL4 was isolated using the technique published by Chomczynski and Sacchi (1987). Probe-protection measurements of IL-2 mRNA using the 430-nt IL-2 probe have been described elsewhere (McGuire and Rothenberg, 1987; McGuire et al., 1988). The average number of molecules per cell was determined using a Phosphor Imager (Molecular Dynamics, Sunnyvale, California) and assuming complete recovery of protected products from each sample of $10^6$ cell equivalents. The sensitivity of this technique was 0.2 copies per cell (or a total of $1\times10^5$ copies per RNA sample isolated from $10^6$ cells; data not shown), and no IL-2 mRNA was detected in uninduced samples.

### ACKNOWLEDGMENTS

We are very grateful to Lisa Scherer and Paul Boyer for their helpful comments on the manuscript; to John Snyder for help with Fig. 6; to Robin Condie for excellent care of the SCID mice; to David Anderson, Eric Davidson, Peter Dervan, and William Dreyer for allowing us access to critical instrumentation used in this project; and to Cathy Blagg, for expert help in typing the manuscript. This work was supported by NIH grant AI19752.

*(Received August 21, 1992)*

*(Accepted November 23, 1992)*

### REFERENCES

Allison J.P., and Havran W.L. (1991). The immunobiology of T cells with invariant γδ antigen receptors. Annu. Rev. Immunol. **9:** 679–705.

Bogen S.A., Weinberg D.S., and Abbas A.K. (1991). Histologic analysis of T lymphocyte activation in reactive lymph nodes. J. Immunol. **147:** 1537–1541.

Bosma M.J., and Carroll A.M. (1991). The *scid* mouse mutant: Definition, characterization, and potential uses. Annu. Rev. Immunol. **9:** 323–350.

Boyer P.D., Diamond R.A., and Rothenberg E.V. (1989). Changes in inducibility of IL-2 receptor α-chain and T-cell receptor expression during thymocyte differentiation in the mouse. J. Immunol. **142:** 4121–4130.

Carding S.R., Jenkinson E.J., Kingston R., Hayday A.C., Bottomly K., and Owen J.J.T. (1989). Developmental control of lymphokine gene expression in fetal thymocytes during T cell ontogeny. Proc. Natl. Acad. Sci. USA **86:** 3342–3345.

Carding S.R., Keyes S., Jenkinson E.J., Kingston R., Bottomly K., Owen J.J.T., and Hayday A. (1990). Developmentally regulated fetal thymic and extrathymic T-cell receptor gene expression. Genes & Devel. **4:** 1304–1315.

Chen D., and Rothenberg E.V. (1993). Molecular basis for developmental changes in interleukin-2 inducibility. Mol. Cell. Biol. **13:** 228–237.

Chomczynski P., and Sacchi N. (1987). Single-step method of RNA isolation by acid guanidinium thiocyanate-phenol-chloroform extraction. Anal. Biochem. **162:** 156–159.

Farr A., Hosier S., Nelson A., Itohara S., and Tonegawa S. (1990). Distribution of thymocytes expressing $\gamma\delta$ receptors in the murine thymus during development. J. Immunol. **144:** 492–498.

Fischer M., McNeil I., Suda T., Cupp J.E., Shortman K.E., and Zlotnik A. (1991). Cytokine production by immature thymocytes. J. Immunol. **146:** 3452–3456.

Havran W.L., and Allison J.P. (1988). Developmentally ordered appearance of thymocytes expressing different T-cell antigen receptors. Nature **335:** 443–445.

Howe R.C., and MacDonald H.R. (1988). Heterogeneity of immature (Lyt-2$^-$/L3T4$^-$) thymocytes: Identification of four major phenotypically distinct subsets differing in cell cycle status and *in vitro* activation requirements. J. Immunol. **140:** 1047–1055.

Ikuta K., Kina T., MacNeil I., Uchida N., Peault B., Chien Y.-H., and Weissman I.L. (1990). A developmental switch in thymic lymphocyte maturation potential occurs at the level of hematopoietic stem cells. Cell **62:** 863–874.

Ishida Y., Nishi M., Taguchi O., Inaba K., Hattori M., Minato N., Kawaichi M., and Honjo T. (1989). Expansion of natural killer cells but not T cells in human interleukin 2/interleukin 2 receptor (Tac) transgenic mice. J. Exp. Med. **170:** 1103–1115.

Jenkinson E.J., Kingston R., and Owen J.J.T. (1987). Importance of IL-2 receptors in intrathymic generation of cells expressing T-cell receptors. Nature **329:** 160–162.

Kisielow P., Krammer P.H., Hultner L., and von Boehmer H.R. (1985). Maturation of fetal thymocytes in organ culture; secretion of interleukin-2, interferon and colony stimulating factors. Thymus **7:** 189–198.

Kroemer G., de Cid R:, de Alboran I.M., Gonzalo J.-A., Iglesias A., Martinez-A C., and Gutierrez-Ramos J.C. (1991). Immunological self-tolerance: An analysis employing cytokines or cytokine receptors encoded by transgenes or a recombinant vaccinia virus. Immunol. Rev. **122:** 173–204.

LeClerq G., DeSmedt M., Tison B., and Plum J. (1990). Preferential expansion of T-cell receptor V$\gamma$3-positive cells in IL-2 stimulated fetal thymocytes. J. Immunol. **145:** 3992–3997.

Lindsten T., June C.H., Ledbetter J.A., Stella G., and Thompson G.B. (1989). Regulation of lymphokine message RNA stability by a surface-mediated T-cell activation pathway. Science **244:** 339–343.

McGuire K.L., and Rothenberg E.V. (1987). Inducibility of interleukin-2 RNA expression in individual mature and immature T lymphocytes. EMBO J. **6:** 939–946.

McGuire K.L., Yang J.A., and Rothenberg E.V. (1988). Influence of activating stimulus on functional phenotype: Interleukin 2 mRNA accumulation differentially induced by ionophore and receptor ligands in subsets of murine T cells. Proc. Natl. Acad. Sci. USA **85:** 6503–6507.

Miller J., Malek T.R., Leonard W.J., Greene W.C., Shevach E., and Germain R.N. (1985). Nucleotide sequence and expression of a mouse interleukin 2 receptor cDNA. J. Immunol. **134:** 4212–4218.

Montgomery R.A., and Dallman M.J. (1991). Analysis of cytokine gene expression during fetal thymic ontogeny using the polymerase chain reaction. J. Immunol. **147:** 554–560.

Penit C. (1986). *In vivo* thymocyte maturation. BUdR labeling of cycling thymocytes and phenotypic analysis of their progeny support the single lineage model. J. Immunol. **137:** 2115–2121.

Plum J., and de Smedt M. (1988). Differentiation of thymocytes in fetal organ culture: Lack of evidence for the functional role of the interleukin 2 receptor expressed by prothymocytes. Eur. J. Immunol. **18:** 795–799.

Rodewald H.-R., Moingeon P., Lucich J.L., Dosiou C., Lopez P., and Reinherz E.L. (1992). A population of early fetal thymocytes expressing Fc RII/III contains precursors of T lymphocytes and natural killer cells. Cell **69:** 139–150.

Rothenberg E.V. (1992). The development of functionally responsive T cells. Adv. Immunol. **51:** 85–214.

Rothenberg E.V., McGuire K.L., and Boyer P.D. (1988). Molecular indices of functional competence in developing T cells. Immunol. Rev. **104:** 29–53.

Rothenberg E.V., Diamond R.A., Pepper K.A., and Yang J.A. (1990). IL-2 gene inducibility in T cells before T cell receptor expression: Changes in signaling pathways and gene expression requirements during intrathymic maturation. J. Immunol. **144:** 1614–1624.

Schorle H., Holtschke T., Hunig T., Schimpl A., and Horak I. (1991). Development and function of T cells in mice rendered interleukin-2 deficient by gene targeting. Nature **352:** 621–624.

Shaw J., Meerovitch K., Bleackley R.C., and Paetkau V. (1988). Mechanisms regulating that level of IL-2 mRNA in T lymphocytes. J. Immunol. **140:** 2243–2248.

Shores E.W., Sharrow S.O., Uppenkamp I., and Singer A. (1990). T-cell receptor-negative thymocytes from *scid* mice can be induced to enter the CD4/CD8 differentiation pathway. Eur. J. Immunol. **20:** 69–77.

Shores E.W., van Ewijk W., and Singer A. (1991). Disorganization and restoration of thymic medullary epithelial cells in T-cell receptor-negative *scid* mice: Evidence that receptor-bearing lymphocytes influences maturation of the thymic microenvironment. Eur. J. Immunol. **21:** 1657–1661.

Skinner M., LeGros G., Marbrook J., and Watson J.D. (1987). Development of fetal thymocytes in organ cultures. Effect of interleukin-2. J. Exp. Med. **165:** 1481–1493.

Tentori L., Longo D.L., Zuñiga-Pflücker J.C., Wang C., and Kruisbeek A.M. (1988a). Essential role of the interleukin 2-interleukin 2 receptor pathway in thymocyte maturation *in vivo*. J. Exp. Med. **168:** 1741–1747.

Tentori L., Pardoll D.M., Zuñiga-Pflücker J.C., Hu-Li J., Paul W.E., Bluestone J.A., and Kruisbeek A.M. (1988b). Proliferation and production of IL-2 and B cell stimulatory factor/IL-4 in early fetal thymocytes by activation through Thy-1 and CD3. J. Immunol. **140:** 1089–1094.

Van Ewijk W. (1991). T-cell differentiation is influenced by thymic microenvironments. Annu. Rev. Immunol. **9:** 591–615.

Waanders G.A. (1990). Modulation of T-cell differentiation by cytokine and monoclonal antibody treatment of fetal thymic organ culture. Ph.D. thesis, Monash University.

Waanders G.A., and Boyd R.L. (1990). The effects of interleukin 2 on early and late thymocyte differentiation in foetal thymus organ culture. Int. Immunol. **2:** 461–468.

Zuñiga-Pflücker J.C., Smith K.A., Tentori L., Pardoll D.M., Longo D.L., and Kruisbeek A.M. (1990). Are the IL-2 receptors expressed in the murine fetal thymus functional? Devel. Immunol. **1:** 59–66.

Zuñiga-Pflücker J.C., and Kruisbeek A.M. (1990). Intrathymic radioresistant stem cells follow an IL-2/IL-2R pathway during thymic regeneration after sublethal irradiation. J. Immunol. **144:** 3736–3740.

